# Air toxics and birth defects: a Bayesian hierarchical approach to evaluate multiple pollutants and spina bifida

**DOI:** 10.1186/1476-069X-14-16

**Published:** 2015-02-09

**Authors:** Michael D Swartz, Yi Cai, Wenyaw Chan, Elaine Symanski, Laura E Mitchell, Heather E Danysh, Peter H Langlois, Philip J Lupo

**Affiliations:** Division of Biostatistics, University of Texas School of Public Health, Houston, TX USA; Division of Epidemiology, Human Genetics and Environmental Sciences, University of Texas School of Public Health, Houston, TX USA; Department of Pediatrics, Section of Hematology-Oncology and Texas Children’s Cancer Center, Baylor College of Medicine, Houston, TX USA; Birth Defects Epidemiology and Surveillance Branch, Texas Department of State Health Services, Austin, TX USA

**Keywords:** Bayesian hierarchical models, Birth defects, Hazardous air pollutants, Maternal exposure, Multi-pollutant, Spina bifida

## Abstract

**Background:**

While there is evidence that maternal exposure to benzene is associated with spina bifida in offspring, to our knowledge there have been no assessments to evaluate the role of multiple hazardous air pollutants (HAPs) simultaneously on the risk of this relatively common birth defect. In the current study, we evaluated the association between maternal exposure to HAPs identified by the United States Environmental Protection Agency (U.S. EPA) and spina bifida in offspring using hierarchical Bayesian modeling that includes Stochastic Search Variable Selection (SSVS).

**Methods:**

The Texas Birth Defects Registry provided data on spina bifida cases delivered between 1999 and 2004. The control group was a random sample of unaffected live births, frequency matched to cases on year of birth. Census tract-level estimates of annual HAP levels were obtained from the U.S. EPA’s 1999 Assessment System for Population Exposure Nationwide. Using the distribution among controls, exposure was categorized as high exposure (>95^th^ percentile), medium exposure (5^th^-95^th^ percentile), and low exposure (<5^th^ percentile, reference). We used hierarchical Bayesian logistic regression models with SSVS to evaluate the association between HAPs and spina bifida by computing an odds ratio (OR) for each HAP using the posterior mean, and a 95% credible interval (CI) using the 2.5^th^ and 97.5^th^ quantiles of the posterior samples. Based on previous assessments, any pollutant with a Bayes factor greater than 1 was selected for inclusion in a final model.

**Results:**

Twenty-five HAPs were selected in the final analysis to represent “bins” of highly correlated HAPs (*ρ* > 0.80). We identified two out of 25 HAPs with a Bayes factor greater than 1: quinoline (OR_high_ = 2.06, 95% CI: 1.11-3.87, Bayes factor = 1.01) and trichloroethylene (OR_medium_ = 2.00, 95% CI: 1.14-3.61, Bayes factor = 3.79).

**Conclusions:**

Overall there is evidence that quinoline and trichloroethylene may be significant contributors to the risk of spina bifida. Additionally, the use of Bayesian hierarchical models with SSVS is an alternative approach in the evaluation of multiple environmental pollutants on disease risk. This approach can be easily extended to environmental exposures, where novel approaches are needed in the context of multi-pollutant modeling.

**Electronic supplementary material:**

The online version of this article (doi:10.1186/1476-069X-14-16) contains supplementary material, which is available to authorized users.

## Background

Birth defects affect approximately 6% of births worldwide [1]. In the United States (U.S.), birth defects are the leading cause of pediatric hospitalizations [2], medical expenditures [3], and death in the first year of life [4]. Neural tube defects (NTDs), one of the most common groups of birth defects, are complex malformations of the central nervous system that result from failure of neural tube closure [1]. One of the most common NTDs is spina bifida. Infants with spina bifida experience both increased morbidity and mortality compared to their unaffected contemporaries [5, 6]. Although these defects are clinically significant, little is known about their etiology. However, there is growing evidence that these conditions are associated with maternal exposure to environmental toxicants [7].

The U.S. Clean Air Act of 1990 classified 188 environmental toxicants as air toxics or hazardous air pollutants (HAPs). In 1999, the United States Environmental Protection Agency (U.S. EPA) went on to identify 33 HAPs that present the greatest threat to public health [8]. Included in this list are: aromatic solvents (e.g., benzene), chlorinated solvents (e.g., methylene chloride) and metals (e.g., nickel compounds). HAPs are a particularly important group of environmental toxicants because: 1) they are known or suspected to cause a range of adverse health outcomes [9]; 2) their levels are increasing in communities throughout the U.S. [10–12]; and 3) there are currently no national air quality standards for HAPs, as there are for the criteria air pollutants (e.g., carbon monoxide and ozone) [13].

While there is evidence that maternal exposure to benzene is associated with spina bifida in offspring [14], to our knowledge there have been no assessments to evaluate the role of multiple HAPs simultaneously on the risk of spina bifida or other birth defects. When simultaneously evaluating multiple predictors for disease outcome, current methods focus on building multivariable models, rather than the evaluation of single exposures adjusting for known covariates [15]. Yet traditional stepwise methods for model selection using statistics computed at each step can lead to biased estimates [15]. Bayesian variable selection techniques, such as stochastic search methods, offer a solution to this problem. Specifically, stochastic search methods include model selection uncertainty in the model building process to provide more comprehensive information regarding important predictors [16–18]. These stochastic search methods, also considered a Bayesian hierarchical mixture model, can jointly model multiple factors while including estimates of uncertainty to balance power and false discovery control [18, 19]. Specifically, simulations have shown that priors can be selected such that the evidence of a correct association is higher for stochastic search methods compared to stepwise regression methods when selecting a model [18]. Stochastic search methods also perform well in situations with correlated predictors (r^2^ ≈ 0.25-0.80) [17–20]. As a result, stochastic search variable selection methods have been successfully employed when investigating complex diseases, especially when assessing multiple genetic predictors [21, 22]. In the current study, we evaluated the association between maternal exposure to the 33 HAPs identified by the U.S. EPA and spina bifida in offspring using hierarchical Bayesian modeling that includes stochastic search.

## Methods

### Study population

The study population has been described previously [14]. Briefly, data on live births, stillbirths, and electively terminated fetuses with NTDs (including spina bifida) delivered between January 1, 1999 and December 31, 2004 were obtained from the Texas Birth Defects Registry (*n* = 1,108). The registry is a population-based, active surveillance system that has monitored births, fetal deaths, and terminations throughout the state since 1999. A stratified random sample of unaffected live births delivered in Texas between January 1, 1999 and December 31, 2004 was selected as the control group using a ratio of 4 controls to 1 case. Controls were frequency matched to cases by year of birth due to the decreasing birth prevalence of NTDs over time [23]. This yielded a group of 4,132 controls. The study protocol was reviewed and approved by the Institutional Review Boards of the Texas Department of State Health Services, The University of Texas Health Science Center at Houston, and Baylor College of Medicine.

### Exposure assessment

Census tract-level estimates of ambient HAP concentrations were obtained from the U.S. EPA’s 1999 Assessment System for Population Exposure Nationwide (ASPEN) [24–26]. The methods used for ASPEN have been described fully elsewhere [25, 26]. Briefly, ASPEN is part of the National Air Toxic Assessment [12] and is based on the U.S. EPA’s Industrial Source Complex Long Term Model. It takes into account emissions data, rate, location, and height of pollutant release; meteorological conditions; and the reactive decay, deposition, and transformation of pollutants. Ambient air levels of HAPs are reported as annual concentrations in μg/m^3^[26]. Residential HAP levels were estimated based on maternal address at delivery as reported on vital records for cases and controls. Addresses were geocoded and mapped to their respective census tracts by the Texas Department of State Health Services. Our data included mothers from 2,381 census tracts.

### Covariates

The following covariates were selected *a priori*[14, 27–31] as potential confounders and were obtained or calculated from vital records data: infant sex; year of birth; maternal race/ethnicity (non-Hispanic white, non-Hispanic black, Hispanic, or other); maternal birth place (U.S., Mexico, or other); maternal age (<20, 20–24, 25–29, 30–34, 35–39, or ≥40 years); maternal education (<high school, high school, or > high school); marital status (married or not married); parity (0, 1, 2, or ≥3); maternal smoking (no or yes); and season of conception (spring, summer, fall, or winter). Additionally, as the exposure assessment was based on census tract-level estimates, we opted to include a census tract-level estimate of socioeconomic status (percent of households below the poverty level), which was obtained from the U.S. Census 2000 Summary File 3. The percent of households in each census tract below the poverty level was categorized into quartiles (low, medium-low, medium-high, and high poverty level), based on the distribution among the controls.

### Statistical analysis

Descriptive statistics included distributional characteristics of the 33 HAPs and frequency distributions for demographic variables stratified on case–control status. Differences in the distribution of categorical variables between cases and controls were determined using chi-squared tests where *P* < 0.05. Correlations between HAPs were determined using Spearman’s rank correlation. Because SVSS is most appropriate when variables are correlated (i.e., *ρ* = 0.25-0.80) but not highly correlated (i.e., *ρ* > 0.80), we grouped (or “binned”) HAPs with high correlation (i.e., *ρ* > 0.80) and selected pollutants to represent a given bin based on existing science and correlations with the other HAPs within the bin. Omitting variables based on correlation and existing scientific evidence is a reasonable approach to reduce multicollinearity [32]. To bin the HAPs, we used an algorithm based on correlation that is commonly used in genetic association studies [33]. Once the bins were defined, we selected HAPs within the bins to either best represent the bin (maximum correlation with other HAPS), or a combination of highest correlation and existing evidence of association with birth defects.

Two primary association analyses were conducted. First, we examined the association between maternal exposure to each HAP individually and spina bifida in offspring, adjusting for year of birth, maternal education, maternal race/ethnicity, maternal smoking, and census tract poverty status [14] using Bayesian hierarchical logistic regression. Second, we performed a multi-pollutant analysis using Bayesian hierarchical logistic regression combined with Stochastic Search Variable Selection (SSVS) to jointly investigate all HAPs while adjusting for the same covariates. The Bayesian hierarchical model can be interpreted as a mixed-effects logistic model as it provides a fixed-effect for the association between maternal exposure to each HAP and spina bifida in offspring, as well as a random intercept to account for the within-group correlation resulting from the use of a census tract-level exposure assignment [34]. SSVS adds a coherent data driven probabilistic framework to search through the fixed effects and identify potentially important associations [16–18, 35]. Additionally, the simultaneous inference of multiple HAPs in a Bayesian framework is not affected by multiple comparisons in the same way as in a frequentist framework, and can easily be accommodated using an appropriate prior [36, 37]. For a comparison to SSVS, we also performed a standard hierarchical Bayesian model without selection, assessing the HAPs simultaneously.

We categorized the HAPs into three categories based on their distribution among the controls: high exposure (above the 95^th^ percentile of controls), medium exposure (between the 5^th^ percentile to 95^th^ percentile of controls), and low exposure (below the 5^th^ percentile, used as the reference) [14, 38]. As a sensitivity analysis to our categorization, the Bayesian analysis was repeated modeling each HAP as a continuous measure. Due to the large variation in concentrations across all HAPs, we centered and standardized each pollutant.

*Prior Distributions*: For the individual pollutant analysis, we used a hierarchical prior for the random intercept based on previously established methods [34]. Specifically, the random intercept was given a normal prior distribution with mean of 0, and the standard deviation component for the random intercept was given a uniform hyper prior on the range of 0 to 3. The priors for the covariate fixed effects were normally distributed with a mean of 0 and variance of 10. In the context of logistic regression, these priors are considered non-informative. In the multi-pollutant model with SSVS, we used the same priors for the random and fixed parameters for the covariates. For the parameters corresponding to the HAPs, we assumed a mixture prior for SSVS [18]. This mixture involves a normal distribution with mean 0 and variance 0.001 if the variable was not selected, and a normal prior with mean 0 and variance 10 if the variable was selected. Through selection of 0.001 as the variance for the prior when the variable was not selected, the null OR is defined as being in the interval from 0.97 to 1.03 with a 99% probability. In other words, we consider an OR in this interval to not be meaningfully different from a null association [19]. We set the prior probability of inclusion for each variable to 0.25, and sensitivity analyses were conducted using a prior probability of inclusion of 0.50. These settings for prior probabilities for inclusion have been shown to have a good balance between power and false positives [18]. Each covariate (including those that were categorized) had an independent mixture prior.

*Model Estimation and Selection*: We estimated the posterior distributions of the hierarchical Bayesian models using Markov chain Monte Carlo (MCMC) methods. For the single pollutant analyses, we computed an OR for each pollutant using the posterior mean, and a 95% credible interval (CI) using the 2.5^th^ and 97.5^th^ quantiles of the posterior samples [39]. For the multi-pollutant model using SSVS, we computed the marginal Bayes factor for each pollutant [40]. In brief, the marginal Bayes factor is a ratio of the prior odds to the posterior odds that summarizes the evidence for selection of each variable, given the data. Therefore, any Bayes factor greater than 1 implies some evidence for inclusion in the model, with values much greater than 1 representing stronger evidence [41, 42]. We included those HAPs with a marginal Bayes factor greater than 1 in a joint model, computed the joint posterior distribution of the selected model through MCMC, and computed the OR and 95% CI for the joint model using the posterior mean and quantiles of the beta coefficients as described. For categorical covariates, if either high or medium were selected, we considered both as selected for final model estimation. For all MCMC computations, we simulated two chains with separate initial values, each consisting of 150,000 iterations. We discarded the first 50% of each chain as burn in to allow the chain to converge to the posterior distribution. We assessed convergence through how well the posterior means for all parameters correlated between the two chains. We considered a correlation higher than 0.95 indicating that the chains had sufficiently converged. Once convergence was determined to be adequate, we pooled the retained iterations to compute our estimates of the OR and 95% CI. All MCMC computations were performed using WinBUGS 1.4 [43], and posterior inference was performed using R (64 bit v. 3.0.2).

## Results

The distributional characteristics of 32 of the 33 U.S. EPA-designated HAPs based on the 1999 ASPEN model are presented in Table 1. Coke oven emissions, which were included in the list of 33, were not estimated for Texas in the 1999 ASPEN model. There were four groups of highly correlated HAPs (Table 2). As noted, we identified one or two HAPs from each group to represent that “bin” of HAPs based on selection criteria used in genetic association studies for highly correlated single nucleotide polymorphisms [33]. Specifically, benzene and methylene chloride were selected to represent the highly correlated group consisting of acetaldehyde, acrolein, and formaldehyde, benzene and methylene chloride. 1,1,2,2-Tetrachloroethane was selected to represent the highly correlated group including ethylene dibromide, propylene dichloride, and 1,1,2,2-tetrachloroethane. Vinyl chloride was selected to represent the highly correlated group including ethylene dichloride and vinyl chloride. Diesel particulate matter was selected to represent the highly correlated group consisting of diesel particulate matter and nickel compounds. After applying these criteria, 25 HAPs remained in our analysis.

**Table 1:** **Distributional characteristics of hazardous air pollutants (μg/m** ^**3**^ **) based on the 1999 U.S. EPA ASPEN Model, Texas**

Pollutant	Mean	Median	5 ^th^ percentile	25 ^th^ percentile	75 ^th^ percentile	95 ^th^ percentile
Acetaldehyde	1.50	1.45	0.62	0.86	1.93	2.97
Acrolein	0.11	0.09	0.01	0.04	0.14	0.32
Acrylonitrile	4.08 × 10^-03^	1.80 × 10^-04^	4.18 × 10^-06^	4.00 × 10^-05^	7.80 × 10^-04^	2.41 × 10^-02^
Arsenic compounds	6.00 × 10^-05^	2.00 × 10^-05^	1.18 × 10^-06^	1.00 × 10^-05^	5.00 × 10^-05^	1.40 × 10^-04^
Benzene	1.40	1.26	0.45	0.85	1.73	2.83
Beryllium compounds	1.00 × 10^-05^	1.00 × 10^-05^	4.53 × 10^-07^	2.06 × 10^-06^	1.00 × 10^-05^	2.00 × 10^-05^
1,3-Butadiene	0.13	0.12	0.01	0.07	0.17	0.30
Cadmium compounds	1.00 × 10^-04^	2.00 × 10^-05^	8.08 × 10^-07^	4.52 × 10^-06^	5.00 × 10^-05^	2.20 × 10^-04^
Carbon tetrachloride	0.28	0.27	0.27	0.27	0.27	0.29
Chloroform	0.08	0.07	0.04	0.05	0.09	0.16
Chromium VI	3.40 × 10^-04^	7.00 × 10^-05^	2.65 × 10^-06^	2.00 × 10^-05^	2.10 × 10^-04^	1.42 × 10^-03^
1,3-Dichloropropene	0.08	0.07	0.01	0.04	0.11	0.17
Diesel particulate matter	1.18	0.97	0.28	0.53	1.42	2.81
Ethylene oxide	1.19 × 10^-02^	8.25 × 10^-03^	7.80 × 10^-04^	4.35 × 10^-03^	1.42 × 10^-02^	3.38 × 10^-02^
Ethylene dibromide	2.57 × 10^-02^	2.98 × 10^-02^	4.00 × 10^-04^	1.93 × 10^-02^	3.58 × 10^-02^	3.73 × 10^-02^
Ethylene dichloride	0.05	0.05	0.01	0.03	0.05	0.10
Formaldehyde	1.60	1.57	0.55	1.02	2.01	2.97
Hexachlorobenzene	3.00 × 10^-05^	4.15 × 10^-07^	1.08 × 10^-07^	2.38 × 10^-07^	1.08 × 10^-06^	6.00 × 10^-06^
Hydrazine	1.00 × 10^-05^	2.74 × 10^-09^	2.88 × 10^-15^	2.31 × 10^-10^	6.14 × 10^-08^	4.00 × 10^-05^
Lead compounds	2.49 × 10^-03^	1.73 × 10^-03^	7.00 × 10^-05^	4.90 × 10^-04^	3.07 × 10^-03^	7.02 × 10^-03^
Manganese compounds	1.50 × 10^-03^	6.40 × 10^-04^	1.00 × 10^-05^	1.00 × 10^-04^	1.37 × 10^-03^	4.60 × 10^-03^
Mercury compounds	1.63 × 10^-03^	1.52 × 10^-03^	1.50 × 10^-03^	1.51 × 10^-03^	1.57 × 10^-03^	1.99 × 10^-03^
Methylene chloride	0.49	0.48	0.11	0.33	0.63	0.88
Nickel compounds	7.30 × 10^-04^	4.10 × 10^-04^	2.00 × 10^-05^	1.30 × 10^-04^	8.00 × 10^-04^	2.39 × 10^-03^
Polychlorinated biphenyls	4.00 × 10^-04^	3.90 × 10^-04^	3.80 × 10^-04^	3.80 × 10^-04^	4.00 × 10^-04^	4.70 × 10^-04^
Perchloroethylene	0.20	0.20	0.02	0.12	0.27	0.38
Polycyclic Organic Matter	7.73 × 10^-03^	6.58 × 10^-03^	1.55 × 10^-03^	4.07 × 10^-03^	9.63 × 10^-03^	1.78 × 10^-02^
Propylene Dichloride	2.15 × 10^-02^	2.42 × 10^-02^	6.64 × 10^-03^	1.80 × 10^-02^	2.77 × 10^-02^	2.80 × 10^-02^
Quinoline	1.00 × 10^-05^	2.70 × 10^-08^	9.65 × 10^-14^	4.92 × 10^-09^	2.92 × 10^-07^	2.00 × 10^-05^
1,1,2,2-Tetrachloroethane	0.06	0.07	0.02	0.05	0.08	0.08
Trichloroethylene	0.10	0.09	0.05	0.07	0.11	0.16
Vinyl chloride	6.42 × 10^-02^	6.60 × 10^-02^	1.04 × 10^-03^	4.36 × 10^-02^	7.93 × 10^-02^	1.19 × 10^-01^

**Table 2:** **Hazardous air pollutants with correlations greater than** ***ρ*** **> 0.80, Texas, 1999**

Pollutant	Acet	Acro	Form	Benz	MCl	EDbr	PD	TCE	EDcl	VCl	DPM	Ni
Acet	1.00	0.97	0.98	0.85	0.79							
Acro	0.97	1.00	0.95	0.81	0.68							
Form	0.98	0.95	1.00	0.89	0.82							
Benz^1^	0.85	0.81	0.89	1.00	0.74							
MCl^1^	0.79	0.68	0.82	0.74	1.00							
EDbr						1.00	0.98	1.00				
PD						0.98	1.00	0.99				
TCE^2^						1.00	0.99	1.00				
EDcl									1.00	0.98		
VCl^3^									0.98	1.00		
DPM^4^											1.00	0.87
Ni											0.87	1.00

*Abbreviations: Acet acetaldyhyde, *Acro* acrolein, *Form* formaldehyde, *Benz* benzene, *MCl* methylene chloride, *EDbr* ethylene dibromide, *PD* propylene dichloride, *TCE* 1,1,2,2-Tetrachloroethane, *EDcl* ethylene dichloride, *VCl* vinyl chloride, *DPM* diesel particulate matter, *Ni* nickel compounds.

^1^Benzene and methylene chloride selected to represent the highly correlated group including acetaldehyde, acrolein and formaldehyde.

^2^1,1,2,2-Tetrachloroethane selected to represent the highly correlated group including ethylene dibromide and propylene dichloride.

^3^Vinyl Chloride selected to represent the highly correlated group including ethylene dichloride.

^4^Diesel particulate matter selected to represent the highly correlated group including nickel compounds.

To minimize etiologic heterogeneity within the case group, cases with an associated chromosomal abnormality or other syndrome (*n* = 75), those with a closed defect (i.e., lipomyelomeningocele, *n* = 88), and those with anencephaly (*n* = 351) were excluded. Cases with missing geocoded maternal address were excluded (*n* = 61). After these exclusions, 533 spina bifida cases were available for analysis. Of the 4,132 controls, 437 were excluded due to missing geocoded maternal address. The final control group consisted of 3,695 unaffected births for analysis. The proportion of case and control mothers missing address information was similar (11.4% and 10.5%, respectively), and there were no significant differences on demographic factors between those with and without a maternal address at delivery. The characteristics of cases and controls are presented in Table 3. Mothers of spina bifida cases were more likely to be Hispanic and to have been born in Mexico compared to mothers of controls (p = 0.003 and p = 0.05, respectively). Additionally, mothers of cases were more likely to live in census tracts with higher poverty levels (p = 0.02). Cases and controls did not significantly differ on other demographic characteristics.

**Table 3:** **Characteristics of spina bifida cases and controls, Texas, 1999-2004**

Characteristic	Controls	Cases	P-value
	***N*** = 3,695	***N*** = 533	
Sex of infant			
Female	1,828 (49.5)	251 (47.3)	0.34
Male	1,867 (50.5)	280 (52.7)	
Maternal race/ethnicity			
Non-Hispanic white	1,344 (36.5)	191 (36.0)	<0.01
Non-Hispanic black	430 (11.7)	54 (10.2)	
Hispanic	1,773 (48.1)	280 (52.8)	
Other	138 (3.7)	5 (0.9)	
Maternal birthplace			
United States	2,592 (70.4)	355 (67.4)	0.05
Mexico	785 (21.3)	145 (27.5)	
Other	306 (8.3)	27 (5.1)	
Maternal age (years)			
<20	501 (13.6)	76 (14.3)	0.32
20-24	1,099 (29.7)	158 (29.6)	
25-29	966 (26.1)	141 (26.5)	
30-34	754 (20.4)	119 (22.3)	
35-39	323 (8.7)	31 (5.8)	
≥40	52 (1.4)	8 (1.5)	
Maternal education			
<High school	1,155 (31.7)	188 (36.4)	0.06
High school	1,195 (32.8)	169 (32.7)	
>High school	1,292 (35.5)	160 (30.9)	
Parity			
0	1,314 (36.9)	190 (37.7)	0.79
1	1,170 (32.9)	157 (31.2)	
2	679 (19.1)	95 (18.8)	
≥3	396 (11.1)	62 (12.3)	
Maternal smoking			
No	3,447 (93.9)	505 (95.5)	0.15
Yes	225 (6.1)	24 (4.5)	
Census tract poverty level			
Low	922 (25.0)	100 (18.8)	0.02
Medium-low	925 (25.0)	144 (27.0)	
Medium-high	925 (25.0)	137 (25.7)	
High	925 (25.0)	152 (28.5)	
Season of conception			
Spring	807 (24.0)	106 (22.5)	0.45
Summer	798 (23.7)	127 (27.0)	
Fall	876 (26.0)	122 (25.9)	
Winter	887 (26.3)	116 (24.6)	

When evaluating the association between the 32 HAPs and spina bifida in single-pollutant models, 14 of the 32 (44%) had 95% CIs excluding 1.0 for either the medium or high exposure categories (Additional file 1: Table S1). Based on the multi-pollutant analysis among the 25 HAPs, when computing the marginal Bayes factors from the SSVS posterior, we identified two with a Bayes factor greater than or 1 (Table 4): quinoline (OR_high_ = 2.06, 95% CI: 1.11-3.87, Bayes factor = 1.01); and trichloroethylene (OR_medium_ = 2.00, 95% CI: 1.14-3.61, Bayes factor = 3.79). These associations are stronger than those of the covariates (Additional file 1: Table S2), while the 95% CIs are of similar width. The unadjusted ORs overestimate these effects due to uncontrolled confounding (Additional file 1: Table S3). For comparison, the joint model without SSVS only identified the medium level of Trichloroethylene as associated with spina bifida (OR = 5.72, 95% CI: 1.44-24.16, Additional file 1: Table S4). The sensitivity analysis using a prior probability of 0.50 yielded similar results (data not shown). Our analysis using HAPs on the continuous scale selected eight HAPs with a Bayes factor greater than 1, however, all of the 95% CIs included 1.0 (data not shown).

**Table 4:** **Hazardous air pollutants associated with spina bifida identified using Stochastic Search Variable Selection (SSVS) with a Bayes factor greater than 1.00**

Pollutant	Pollutant range (μg/m ^3^ )	Cases/Controls	Odds ratio ^1^	95% credible interval	Bayes factor
Quinoline					
Low	<6.50 × 10^-14^	18/176	1.00	Ref.	
Medium	6.50 × 10^-14^ - 1.71 × 10^-5^	434/3149	1.42	(0.87, 2.42)	0.32
High	>1.71 × 10^-5^	39/178	2.06	(1.11, 3.87)	1.01
Trichloroethylene					
Low	<0.0524	13/176	1.00	Ref.	
Medium	0.052 – 0.16	460/3151	2.00	(1.14, 3.61)	3.79
High	>0.16	18/176	1.32	(0.61, 2.80)	0.60

^1^Adjusted for year of birth, maternal education, maternal race/ethnicity, maternal smoking, and census tract poverty status.

## Discussion

To our knowledge, this is the first application of a Bayesian variable selection strategy to evaluate the role of multiple HAPs simultaneously on the risk of birth defects. Overall there is evidence that HAPs may be a significant contributor to the risk of spina bifida. Specifically, in single-pollutant models, a large proportion of HAPs (44%) were positively associated with spina bifida. Additionally, using a Bayesian hierarchical approach with SSVS as a multi-pollutant model, we found two HAPs that were associated with spina bifida: quinoline and trichloroethylene. Mothers who lived in census tracts with high quinolone levels or medium trichloroethylene levels were approximately two times as likely to have a child with spina bifida compared to mothers who lived in census tracts with relatively low levels. The effect estimate for mothers living in census tracts with high levels of trichloroethylene was smaller (OR = 1.32) in comparison to the effect estimate for medium levels (OR = 2.00). This inverted U-shaped dose–response relationship is common among toxicants that act as endocrine disruptors such as trichloroethylene [44–47].

The mechanism by which HAPs may lead to teratogenesis is unknown. However, certain HAPs (e.g., benzene, polycyclic aromatic hydrocarbons) are known to cross the placenta and have been found in cord blood at levels equal to or higher than maternal blood [48]. Potential mechanisms by which HAPs may influence the risk of spina bifida include genetic toxicity and oxidative stress. In fact, these mechanisms may interact to contribute to teratogenesis. Specifically, certain HAPs (e.g., polycyclic aromatic hydrocarbons) can lead to genetic toxicity by covalently binding to DNA. The resulting DNA adducts, if not repaired, are mutagenic, resulting in the disruption of the cell’s microenvironment, which leads to inhibition of important enzymes, cell death, and alteration of other cells [49]. If this occurs during the critical window of embryonic development, the complex cellular processes involved in development may be disturbed, leading to spina bifida. Several HAPs (e.g., benzene, toluene) can also form free radicals known as reactive oxygen species (ROS) [9], which may lead to oxidative stress. These ROS can cause DNA strand breakage or fragmentation leading to cell mutation [49]. The importance of oxidative stress as a mechanism of teratogenesis is suggested by several animal studies [50–55].

Quinoline is a coal tar constituent and is the major tar base in creosote [56]. In mouse models, maternal exposure to quinoline has been shown to induce skeletal and visceral malformations in offspring [57]. Other studies indicate quinoline may cross the placenta into the tissue of the developing fetus [56]. However, to our knowledge, there have been no studies evaluating the association between human maternal exposure to quinoline and the risk of spina bifida or other birth defects, suggesting more work is needed on the potential teratogenicity of this agent.

Most of the trichloroethylene used in the U.S. is released into the atmosphere from industrial degreasing operations [58]. While there is evidence from both animal and human studies that trichloroethylene is associated with birth defects, specifically congenital heart defects [59–61], there is ongoing debate over the teratogenicity of this pollutant [62]. An evaluation using data from Camp Lejeune, North Carolina indicated that mothers exposed to higher levels of trichloroethylene were 2.4 times (95% CI: 0.6-9.6) as likely to have offspring with NTDs compared to those exposed to lower levels [63]. While this association was not statistically significant, the strength of the association was similar to that in our assessment.

While maternal exposure to benzene was associated with spina bifida in the single-pollutant model, it was not selected as a final variable in the multi-pollutant model. The effect estimate for benzene from the single-pollutant Bayesian model for the highly exposed group (OR = 1.99) was similar to that from the previous assessment (OR = 2.30) [14]. The absence of benzene from the final model may be due to multiple factors including: 1) high correlation (ρ > 0.80) with several other HAPs and 2) the estimate of effect was not as strong as other HAPs in the final multi-pollutant model.

Our study must be considered in the light of certain limitations. One potential limitation is the use of modeled predictions of ambient air concentrations of HAPs (i.e., the ASPEN model), which may have resulted in exposure misclassification. However, there is no data source that sufficiently addresses this issue. For instance, personal monitoring is not available on a population scale, and outdoor monitoring in Texas is restricted to certain communities. Therefore, the use of ASPEN data is a cost-effective approach in assessing this important question. An additional potential limitation is the use of ASPEN data from 1999 for the entire study period. It is not recommended by the EPA to include ASPEN data from multiple years simultaneously in one assessment. However, relying on HAP estimates from 1999 alone may be a suitable surrogate for other years as while levels of HAPs are likely to change over time, the relative ranking of census tracts based on ambient levels of HAPs was unlikely to change during the study period [10, 64, 65]. Additionally, ASPEN data have been used in several population-based assessments of adverse health outcomes, including birth defects [14, 65–68]. Lastly, information on address at conception was unavailable, and, therefore, we were limited to basing the exposure assignment on maternal address at delivery. However, our previous work suggests that census tract-level exposure assessment is not significantly different when assessing HAP exposure using ASPEN data between the time of conception and delivery [69].

Another potential limitation is the use of an area-based (census tract-level) measure of HAP exposure. Using area-based measures of exposure always assumes some level of increased exposure misclassification, especially compared to individual-level measures. However, using census-tract level exposure information, as was used in this assessment, lessens the amount of potential exposure misclassification compared to using county-level information, which is commonly used in epidemiologic studies of environmental exposures [47, 70–72]. In addition, it is possible that the amount of exposure misclassification could vary by each HAP included in this assessment, potentially introducing additional exposure measurement error with a complex correlation structure, all of which could bias the effect estimates towards the null.

Another point to discuss is the correlation among HAPs in our data. We chose a correlation binning technique commonly employed in statistical genetics to reduce the correlation of HAPS to be below 0.80 while representing as much of the association information as possible in the HAPs for the model [33]. Other methods could have been used, such as binning pollutants on chemical properties, or perhaps targeted source. However, since the statistical model assesses association through correlations, defining bins of HAPs based on correlation seems to be most congruent with the statistical modeling approach. We added a scientific component in our dimension reduction. Instead of purely using statistics to define the representative of each bin, we chose the representative HAPs for a bin based on 1) previously reported associations as well as 2) the level of correlation of each HAP with the other HAPs in the bin. This approach allowed for the combination of previous scientific evidence as well as statistics to represent each bin.

Previous evidence suggests that SSVS may be prone to favor false negative results [18, 19]. Using a Bayes factor threshold of greater than 1 usually reduces the number of false negatives; however, even in the case of increased false negatives, on average, SSVS methods are more likely to generate correct associations compared to standard selection methods [18].

Strengths of this study include the use of a population-based birth defects registry that employs an active surveillance system to ascertain cases throughout the state of Texas. This should limit the potential for selection bias. Furthermore, the Texas Birth Defects Registry includes information on pregnancy terminations reducing potential bias due to the exclusion of these cases. An additional strength was the use of a relatively small (census tract-level) measure of exposure. Using larger geographic units to estimate exposure (e.g., counties) may not capture the spatial variability of HAPs [73].

An important aspect of this study was the Bayesian hierarchical approach for evaluating multiple pollutants while also accounting for the within-group correlation resulting from the use of a census tract-level exposure assignment through the random intercept [34]. Traditional models based on variable selection in a stepwise approach can lead to biased estimates [15]. Bayesian variable selection techniques (e.g., SVSS) offer an attractive alternative to multi-pollutant modeling. Specifically, SSVS includes model selection uncertainty in the model building process to provide more comprehensive information regarding important predictors [16–18]. In our assessment, the Bayesian hierarchical approach resulted in the selection of two HAPs in the final multivariable model; however, when modeling the association with spina bifida in the single-pollutant models, we detected 14 HAPs with statistically significant associations with spina bifida, some of which may be false positives. When compared with the traditional single pollutant models, the multivariable model reduces the number of detected pollutants from 14 to two.

In conclusion, we believe the use of Bayesian hierarchical models with SSVS provides a robust alternative in the evaluation of multiple environmental pollutants on disease risk as this approach allows the joint assessment of multiple factors while including estimates of uncertainty to balance power and false discovery control [18]. Bayesian methods have been reported to outperform conventional maximum-likelihood-estimation techniques for prediction and are useful in settings where multiple exposures are evaluated [36, 37]. Additionally, concerns about multiple comparisons can be eliminated in the simultaneous assessment of multiple HAPs within a Bayesian framework [36, 37]. Specifically, SSVS type methods may be prone to favoring false negatives [18, 19] (SIM and Devocht), meaning that false positives due to multiple comparisions are not an issue. This approach has been used successfully when assessing the role of multiple genetic variants on complex diseases [18, 21, 22, 74], and can be easily extended to environmental exposures, where novel approaches are needed in the context of multi-pollutant modeling.

## Electronic supplementary material

Additional file 1: Table S1: Associations between estimated hazardous air pollutants and spina bifida using single-pollutant Bayesian hierarchical models. **Table S2.** Associations between each covariate included in the final joint model and spina bifida. **Table S3.** Associations of hazardous air pollutants selected in the final joint model using Stochastic Search Variable Selection (SVSS) and spina bifida: Unadjusted results. **Table S4.** Associations of hazardous air pollutants and spina bifida: Multivariable results without SVSS. (DOCX 30 KB)

## Abbreviations

ASPEN: Assessment System for Population Exposure Nationwide
CI: Credible interval
HAPs: Hazardous air pollutants
OR: Odds ratio
NTDs: Neural tube defects
SSVS: Stochastic Search Variable Selection
U.S. EPA: United States Environmental Protection Agency.
