# Supplementary material for: Air toxics and birth defects: a Bayesian hierarchical approach to evaluate multiple pollutants and spina bifida
Source: Environ Health. 2015 Feb 9;14:16. doi: 10.1186/1476-069X-14-16 (PMC4429479; doi:10.1186/1476-069X-14-16)
Supplement: Supplementary file 1 — Additional file 1: Table S1: Associations between estimated hazardous air pollutants and spina bifida using single-pollutant Bayesian hierarchical models. Table S2. Associations between each covariate included in the final joint model and spina bifida. Table S3. Associations of hazardous air pollutants selected in the final joint model using Stochastic Search Variable Selection (SVSS) and spina bifida: Unadjusted results. Table S4. Associations of hazardous air pollutants and spina bifida: Multivariable results without SVSS. (DOCX 30 KB) [file 12940_2014_852_MOESM1_ESM.docx]

Table S1. Associations between estimated hazardous air pollutants and spina bifida using single-pollutant Bayesian hierarchical models

| Pollutant | Odds Ratio | 95% Credible Interval |
| --- | --- | --- |
| Acetaldehyde |  |  |
| Low | 1.00 | Ref. |
| Medium | 1.44 | (0.94, 2.38) |
| High | 1.68 | (0.95, 3.10) |
| Acrolein |  |  |
| Low | 1.00 | Ref. |
| Medium | 1.35 | (0.85, 2.30) |
| High | 1.60 | (0.85, 3.03) |
| Acrylonitrile |  |  |
| Low | 1.00 | Ref. |
| Medium | 1.29 | (0.80, 2.18) |
| High | 1.67 | (0.87, 3.21) |
| Arsenic Compounds |  |  |
| Low | 1.00 | Ref. |
| Medium | 1.26 | (0.77, 2.13) |
| High | 1.67 | (0.93, 3.06) |
| Benzene |  |  |
| Low | 1.00 | Ref. |
| Medium | 1.55 | (0.94, 2.59) |
| High | 1.99 | (1.09, 3.83) |
| Beryllium Compounds |  |  |
| Low | 1.00 | Ref. |
| Medium | 1.59 | (0.98, 2.67) |
| High | 1.81 | (0.97, 3.46) |
| 1,3-Butadiene |  |  |
| Low | 1.00 | Ref. |
| Medium | 2.05 | (1.12, 3.62) |
| High | 2.06 | (0.98, 4.13) |
| Cadmium Compounds |  |  |
| Low | 1.00 | Ref. |
| Medium | 1.59 | (0.96, 2.66) |
| High | 1.49 | (0.78, 2.90) |
| Carbon Tetrachloride |  |  |
| Low | 1.00 | Ref. |
| Medium | 1.47 | (0.92, 2.37) |
| High | 1.91 | (1.02, 3.47) |
| Chloroform |  |  |
| Low | 1.00 | Ref. |
| Medium | 1.63 | (0.96, 2.66) |
| High | 1.60 | (0.80, 3.06) |
| Chromium VI |  |  |
| Low | 1.00 | Ref. |
| Medium | 1.45 | (0.87, 2.37) |
| High | 2.11 | (1.18, 3.81) |
| 1,3-Dichloropropene |  |  |
| Low | 1.00 | Ref. |
| Medium | 1.77 | (1.01, 3.09) |
| High | 1.92 | (1.00, 3.87) |
| Diesel Particulate Matter |  |  |
| Low | 1.00 | Ref. |
| Medium | 1.55 | (0.93, 2.56) |
| High | 1.67 | (0.89, 3.22) |
| Ethylene Oxide |  |  |
| Low | 1.00 | Ref. |
| Medium | 1.55 | (0.97, 2.57) |
| High | 2.15 | (1.18, 4.00) |
| Ethylene Dibromide |  |  |
| Low | 1.00 | Ref. |
| Medium | 1.52 | (1.00, 2.51) |
| High | 1.97 | (1.11, 3.73) |
| Ethylene Dichloride |  |  |
| Low | 1.00 | Ref. |
| Medium | 1.54 | (0.96, 2.71) |
| High | 1.89 | (1.02, 3.66) |
| Formaldehyde |  |  |
| Low | 1.00 | Ref. |
| Medium | 1.65 | (0.92, 2.75) |
| High | 1.87 | (0.96, 3.64) |
| Hexachlorobenzene |  |  |
| Low | 1.00 | Ref. |
| Medium | 1.22 | (0.79, 2.04) |
| High | 1.27 | (0.69, 2.29) |
| Hydrazine |  |  |
| Low | 1.00 | Ref. |
| Medium | 1.56 | (0.96, 2.73) |
| High | 1.99 | (1.07, 3.85) |
| Lead Compounds |  |  |
| Low | 1.00 | Ref. |
| Medium | 1.17 | (0.73, 1.84) |
| High | 1.05 | (0.55, 1.96) |
| Manganese Compounds |  |  |
| Low | 1.00 | Ref. |
| Medium | 1.64 | (1.01, 2.83) |
| High | 2.07 | (1.07, 4.08) |
| Mercury Compounds |  |  |
| Low | 1.00 | Ref. |
| Medium | 1.18 | (0.75, 1.94) |
| High | 1.53 | (0.87, 2.73) |
| Methylene Chloride |  |  |
| Low | 1.00 | Ref. |
| Medium | 1.58 | (0.98, 2.53) |
| High | 1.37 | (0.70, 2.57) |
| Nickel Compounds |  |  |
| Low | 1.00 | Ref. |
| Medium | 1.48 | (0.96, 2.33) |
| High | 1.70 | (0.90, 3.08) |
| Polychlorinated Biphenyls |  |  |
| Low | 1.00 | Ref. |
| Medium | 1.34 | (0.87, 2.29) |
| High | 1.14 | (0.61, 2.25) |
| Perchloroethylene |  |  |
| Low | 1.00 | Ref. |
| Medium | 1.45 | (0.91, 2.41) |
| High | 1.25 | (0.64, 2.31) |
| Polycyclic Organic Matter |  |  |
| Low | 1.00 | Ref. |
| Medium | 1.09 | (0.71, 1.71) |
| High | 1.14 | (0.61, 2.08) |
| Propylene Dichloride |  |  |
| Low | 1.00 | Ref. |
| Medium | 1.50 | (0.95, 2.47) |
| High | 1.59 | (0.86, 2.99) |
| Quinoline |  |  |
| Low | 1.00 | Ref. |
| Medium | 1.49 | (0.92, 2.37) |
| High | 2.24 | (1.22, 3.99) |
| 1,1,2,2-Tetrachloroethane |  |  |
| Low | 1.00 | Ref. |
| Medium | 1.46 | (0.90, 2.43) |
| High | 1.85 | (1.04, 3.49) |
| Trichloroethylene |  |  |
| Low | 1.00 | Ref. |
| Medium | 2.20 | (1.27, 4.23) |
| High | 1.44 | (0.68, 3.01) |
| Vinyl Chloride |  |  |
| Low | 1.00 | Ref. |
| Medium | 1.54 | (0.97, 2.71) |
| High | 2.15 | (1.19, 4.06) |

Table S2: Associations between each covariate included in the final joint model and spina bifida

| Variable | Odds Ratio | 95% Credible Interval |
| --- | --- | --- |
| Birth Year |  |  |
| 1999 | 1.00 | Ref. |
| 2000 | 0.91 | (0.65, 1.27) |
| 2001 | 0.91 | (0.65, 1.27) |
| 2002 | 0.87 | (0.62, 1.22) |
| 2003 | 0.87 | (0.62, 1.22) |
| 2004 | 1.02 | (0.75, 1.39) |
| Maternal Race |  |  |
| Non-Hispanic White | 1.00 | Ref. |
| Non-Hispanic Black | 0.75 | (0.52, 1.06) |
| Hispanic | 0.90 | (0.70, 1.15) |
| Other | 0.25 | (0.09, 0.58) |
| Maternal Education |  |  |
| Less than High School | 1.00 | Ref. |
| High School | 0.90 | (0.70, 1.15) |
| Greater than High School | 0.89 | (0.68, 1.16) |
| Parity |  |  |
| 0 | 1.00 | Ref. |
| 1 | 0.95 | (0.75, 1.20) |
| 2 | 0.95 | (0.72, 1.25) |
| 3 or more | 1.06 | (0.76, 1.46) |
| Area-level poverty* |  |  |
| Quartile 1 | 1.00 | Ref. |
| Quartile 2 | 1.33 | (1.00, 1.80) |
| Quartile 3 | 1.29 | (0.94, 1.78) |
| Quartile 4 | 1.39 | (1.00, 1.93) |

*Percent of households living below the poverty level at the census tract level

Table S3: Associations of hazardous air pollutants selected in the final joint model using Stochastic Search Variable Selection (SVSS) and spina bifida: Unadjusted results

| Pollutant | Unadjusted Odds Ratio | Crude 95% Credible Interval |
| --- | --- | --- |
| Quinoline |  |  |
| Low | 1.00 | Ref. |
| Medium | 1.24 | (1.07, 1.42) |
| High | 2.13 | (1.42, 3.11) |
| Trichloroethylene |  |  |
| Low | 1.00 | Ref. |
| Medium | 2.95 | (1.93, 4.64) |
| High | 1.69 | (0.77, 3.64) |

Table S4: Associations of hazardous air pollutants and spina bifida: Multivariable results without SVSS

| Pollutant | Odds Ratio | 95% Credible Interval |
| --- | --- | --- |
| Acrylonitrile |  |  |
| Low | 1.00 | Ref. |
| Medium | 0.98 | (0.51, 2.03) |
| High | 1.19 | (0.38, 3.65) |
| Arsenic Compounds |  |  |
| Low | 1.00 | Ref. |
| Medium | 0.84 | (0.37, 1.74) |
| High | 1.28 | (0.41, 3.77) |
| Benzene |  |  |
| Low | 1.00 | Ref. |
| Medium | 1.09 | (0.42, 3.05) |
| High | 2.21 | (0.61, 8.38) |
| Beryllium Compounds |  |  |
| Low | 1.00 | Ref. |
| Medium | 1.10 | (0.46, 2.64) |
| High | 1.08 | (0.34, 3.51) |
| 1,3-Butadiene |  |  |
| Low | 1.00 | Ref. |
| Medium | 1.92 | (0.77, 4.87) |
| High | 1.30 | (0.41, 4.31) |
| Cadmium Compounds |  |  |
| Low | 1.00 | Ref. |
| Medium | 1.53 | (0.65, 3.87) |
| High | 1.02 | (0.36, 3.03) |
| Carbon Tetrachloride |  |  |
| Low | 1.00 | Ref. |
| Medium | 1.14 | (0.54,2.35) |
| High | 1.31 | (0.45, 3.83) |
| Chloroform |  |  |
| Low | 1.00 | Ref. |
| Medium | 0.99 | (0.36, 2.82) |
| High | 0.61 | (0.18, 2.08) |
| Chromium VI |  |  |
| Low | 1.00 | Ref. |
| Medium | 0.67 | (0.24, 1.81) |
| High | 1.00 | (0.28, 3.35) |
| 1,3-Dichloropropene |  |  |
| Low | 1.00 | Ref. |
| Medium | 2.18 | (0.52, 10.02) |
| High | 2.75 | (0.60, 13.37) |
| Diesel Particulate Matter |  |  |
| Low | 1.00 | Ref. |
| Medium | 1.17 | (0.55, 2.47) |
| High | 0.94 | (0.33, 2.66) |
| Ethylene Oxide |  |  |
| Low | 1.00 | Ref. |
| Medium | 0.68 | (0.17, 2.94) |
| High | 0.84 | (0.18, 4.22) |
| Hexachlorobenzene |  |  |
| Low | 1.00 | Ref. |
| Medium | 1.28 | (0.75, 2.27) |
| High | 0.76 | (0.31, 1.86) |
| Hydrazine |  |  |
| Low | 1.00 | Ref. |
| Medium | 1.11 | (0.35, 3.93) |
| High | 0.69 | (0.15, 3.37) |
| Lead Compounds |  |  |
| Low | 1.00 | Ref. |
| Medium | 0.80 | (0.43, 1.51) |
| High | 0.60 | (0.26, 1.36) |
| Manganese Compounds |  |  |
| Low | 1.00 | Ref. |
| Medium | 1.93 | (0.86, 4.69) |
| High | 2.46 | (0.96, 6.81) |
| Mercury Compounds |  |  |
| Low | 1.00 | Ref. |
| Medium | 0.61 | (0.28, 1.42) |
| High | 0.51 | (0.16, 1.53) |
| Methylene Chloride |  |  |
| Low | 1.00 | Ref. |
| Medium | 0.66 | (0.14, 3.33) |
| High | 0.51 | (0.10, 2.82) |
| Polychlorinated Biphenyls |  |  |
| Low | 1.00 | Ref. |
| Medium | 1.28 | (0.76, 2.27) |
| High | 1.08 | (0.53, 2.18) |
| Perchloroethylene |  |  |
| Low | 1.00 | Ref. |
| Medium | 0.45 | (0.13, 1.63) |
| High | 0.38 | (0.09, 1.57) |
| Polycyclic Organic Matter |  |  |
| Low | 1.00 | Ref. |
| Medium | 0.54 | (0.26, 1.13) |
| High | 0.49 | (0.19, 1.26) |
| Quinoline |  |  |
| Low | 1.00 | Ref. |
| Medium | 1.19 | (0.36, 3.60) |
| High | 2.44 | (0.56, 11.06) |
| 1,1,2,2-Tetrachloroethane |  |  |
| Low | 1.00 | Ref. |
| Medium | 0.55 | (0.07, 4.39) |
| High | 0.26 | (0.03, 2.63) |
| Trichloroethylene |  |  |
| Low | 1.00 | Ref. |
| Medium | 5.72 | (1.44, 24.17) |
| High | 3.53 | (0.81, 16.14) |
| Vinyl Chloride |  |  |
| Low | 1.00 | Ref. |
| Medium | 1.64 | (0.25, 8.79) |
| High | 2.74 | (0.29, 24.22) |
